# MiRNA Profiles in Lymphoblastoid Cell Lines of Finnish Prostate Cancer Families

**DOI:** 10.1371/journal.pone.0127427

**Published:** 2015-05-28

**Authors:** Daniel Fischer, Tiina Wahlfors, Henna Mattila, Hannu Oja, Teuvo L. J. Tammela, Johanna Schleutker

**Affiliations:** 1 School of Health Sciences, University of Tampere, 33014 Tampere, Finland; 2 BioMediTech, University of Tampere, and Fimlab Laboratories, Tampere, Finland; 3 Department of Mathematics and Statistics, University of Turku, 20014 Turku, Finland; 4 Department of Urology, Tampere University Hospital and Medical School, University of Tampere, Tampere, Finland; 5 Medical Biochemistry and Genetics, Institute of Biomedicine, University of Turku, Turku, Finland; The University of Hong Kong, CHINA

## Abstract

**Background:**

Heritable factors are evidently involved in prostate cancer (PrCa) carcinogenesis, but currently, genetic markers are not routinely used in screening or diagnostics of the disease. More precise information is needed for making treatment decisions to distinguish aggressive cases from indolent disease, for which heritable factors could be a useful tool. The genetic makeup of PrCa has only recently begun to be unravelled through large-scale genome-wide association studies (GWAS). The thus far identified Single Nucleotide Polymorphisms (SNPs) explain, however, only a fraction of familial clustering. Moreover, the known risk SNPs are not associated with the clinical outcome of the disease, such as aggressive or metastasised disease, and therefore cannot be used to predict the prognosis. Annotating the SNPs with deep clinical data together with miRNA expression profiles can improve the understanding of the underlying mechanisms of different phenotypes of prostate cancer.

**Results:**

In this study microRNA (miRNA) profiles were studied as potential biomarkers to predict the disease outcome. The study subjects were from Finnish high risk prostate cancer families. To identify potential biomarkers we combined a novel non-parametrical test with an importance measure provided from a Random Forest classifier. This combination delivered a set of nine miRNAs that was able to separate cases from controls. The detected miRNA expression profiles could predict the development of the disease years before the actual PrCa diagnosis or detect the existence of other cancers in the studied individuals. Furthermore, using an expression Quantitative Trait Loci (eQTL) analysis, regulatory SNPs for miRNA miR-483-3p that were also directly associated with PrCa were found.

**Conclusion:**

Based on our findings, we suggest that blood-based miRNA expression profiling can be used in the diagnosis and maybe even prognosis of the disease. In the future, miRNA profiling could possibly be used in targeted screening, together with Prostate Specific Antigene (PSA) testing, to identify men with an elevated PrCa risk.

## Introduction

Prostate cancer (PrCa) is the most common noncutaneous malignancy and the second leading cause of cancer-related deaths among men in industrialised countries [1]. In Finland, 4604 new prostate cancer cases were diagnosed in 2012 (Finnish Cancer Registry, http://www.cancer.fi/syoparekisteri/). Aging and PSA testing may be the most evident reasons for the increased number of new cases. The growing incidence creates pressure on the health care system as the concern regarding overtreatment is considerable. Therefore, one of the major challenges is to improve the diagnostic and prognostic tools to be able to distinguish lethal from indolent disease at a curable state of the disease.

The contribution of genetic variants has been studied widely in association with prostate cancer predisposition. Both linkage and GWAS together with the few examples arising from candidate gene approaches have led to the identification of about 100 genetic loci that explain only approximately 30% of the genetic risk for the disease [2] [3] [4] [5]. However, there is no obvious molecular or functional evidence indicating how the variations in these candidate sites or their co-inherited neighbouring variants could cause PrCa. In fact, most of the single nucleotide variants (SNPs) found by GWAS are unlikely to affect the coding sequence of any gene but rather reside in intergenic regions. These findings suggest that they have a regulatory role, such as in transcription, splicing or mRNA stability, instead of a direct effect on the function of the gene product [6].

In recent years, the importance of the non-protein coding genome in the functional regulation of normal development and disease development has become evident. MiRNAs are short non-coding RNAs that regulate their target gene expression typically by binding to the 3’ untranslated region (UTR) of the target mRNA [7]. Individual variation of the miRNA expression levels can influence the expression of the mRNA target gene, causing phenotypic differences.

Several studies have shown that miRNA expression levels are predictive for the outcome of solid tumours and leukaemias, but the contribution of altered miRNA expression levels to genetic cancer susceptibility is not known. The transcriptional activity of protein coding genes is inherited as a quantitative trait, and regulatory polymorphisms associated with the variability in the levels of mRNA are considered to be eQTL. Despite the demonstrated importance, knowledge of the genetic regulation of miRNA expression is still in its infancy. In a recent publication, over one hundred eQTLs in primary fibroblasts were described, indicating at least a partial role for genetic variation in altered miRNA expression [8]. Combined analyses of common SNPs and variations in miRNA expression profiles might serve as one way to elucidate the biological functions of SNPs identified from GWAS in common diseases.

The objective of this study was to evaluate the miRNA expression profiles of lymphoblastoid cell lines (LCL) derived from members of high risk PrCa families. Altered miRNA expression in patient LCLs compared with those from healthy family members provided an opportunity to identify germline variants in promoter or other regulatory regions of protein coding genes as a considerable amount of miRNA expression is correlated to host and target gene expression [9]. The large amount of significant miRNA-wise test results within the data also required the development of a new type of differentially expression analysis pipeline. To develop such a pipeline, differentially expression testing has been combined with the importance measures of the machine learning algorithm, Random Forest [10].

## Materials and Methods

### Ethics Statement

This study has been approved by the respective IRB boards of The Ministry of Social Affairs and Health (SMT), National Supervisory Authority for Welfare and Health (Valvira) and Ethics Committee of Tampere University Hospital. Every individual participating in the study has given written informed consent.

### Study population

All samples are of Finnish origin and the collection of the families has been reported previously [11]. For the miRNA microarray study, 115 cases from 70 PrCa families were used. The selected families had at least two first-degree relatives diagnosed with prostate cancer at any age. Healthy (= no diagnosed prostate cancer) individuals (n = 78) from 47 families were used as the controls. The median age at diagnosis for the cases was 65 (44–86.2) years and the controls had a median age of 57.5 (35.2–83.3) years at the time the samples were obtained.

A subset of individuals (n = 54) from the microarray experiment were genotyped with Illumina’s HumanOmniExpress array for another experiment, and the results are published elsewhere [12]. Hence, those 54 samples could be used here for an eQTL analysis (39 PrCa cases and 15 controls). Additional 83 individuals could be used for validation purposes. Altogether, there were 137 genotyped persons from 33 families (20 overlapping families with the microarray part of the study).

**Fig 1:**
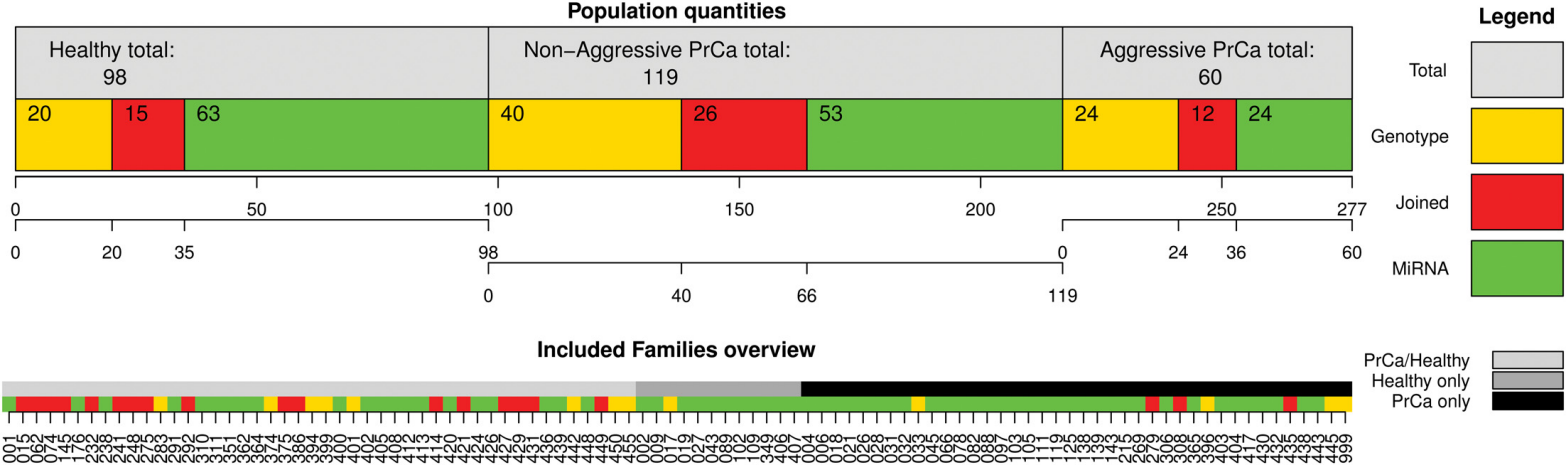
upper: Population quantities, visualisation of how the 277 individuals in this study are distributed among the three health-status groups. For each health group, the number of individuals from the different experiments is shown. The overall number from an experiment is then indicated by the respective coloured box plus the red box (overlap). lower: Visualisation of the familial background. The three options ‘PrCa only’, ‘Healthy only’ or ‘PrCa/Healthy’ are shown and grouped accordingly. Additionally, involvement of different families in the two experiments is shown. Ordering is according to an internal family code.

The clinical outcome of prostate cancer can roughly be classified into aggressive and non-aggressive cancer, based on PSA, Gleason score and other clinical evaluations [13]. Based on these guidelines, the prostate cancer patients from the two experiments were grouped into 36 (36) aggressive and 79 (66) non-aggressive prostate cancers. The maximum number of aggressive cases per family was 3, and the minimum was 1. A detailed overview of the individuals in the study is given in Fig 1.

### RNA extraction from lymphoblastoid cell lines

LCLs were derived by the Epstein-Barr virus transformation of peripheral mononuclear leukocytes from patients and their healthy relatives. The lymphoblastoid cell lines were grown in RPMI-1640 medium (Lonza, Walkersville, MD, USA) supplemented with 10% fetal bovine serum (Sigma-Aldrich, St. Louis, MO, USA) and antibiotics at 37Â°C, 5% CO2 and 95% humidity. The cell pellets were snap-frozen, and total RNA was extracted with Trizol according to the manufacturer’s instructions (Invitrogen, Carlsbad, CA, USA). The RNA yields were quantified using an ND-1000 spectrophotometer (Nanodrop Technologies, Wilmington, DE, USA) and Agilent 2100 Bioanalyzer (Agilent Technologies, Santa Clara, CA, USA).

### MicroRNA microarray analysis

The microRNA expression levels in LCLs were detected using Agilent Human miRNA V2 Oligo Microarray Kit (Agilent Technologies). First, 100 ng of total RNA was used as the starting material, and miRNAs were labeled using the Agilent miRNA Labelling Kit. Labelled RNA was hybridised to Agilent miRNA microarrays that have eight identical arrays per slide, with each array containing probes directed against 817 miRNAs (719 human, 76 non-human viral miRNAs and 22 control miRNAs). In total, 26 slides were used, and the data were extracted using Agilent’s Feature Extraction software (FES), version 10.7.1.1 with the grid layout D_F_20091030. For the data analysis, low quality samples were first removed, resulting in 193 individuals. Each individual Agilent microarray V2 measures 13,737 features, and the FES then used these features to calculate the expression values for 2,466 (2,125 human) probes; based on those probes the 817 miRNA expression values were calculated. The data can be accessed via ArrayExpress accession E-MTAB-3397.

The miRNA expression values are typically calculated with the algorithm *gTotalGeneSignal* as implemented in FES, but in this study, however, probe-wise, background subtracted median values were used instead. The analysis of different probes of the same miRNA as a single miRNA expression value did not appear to be reliable enough, and an analysis at the probe level was more feasible. After calculating the expression values at the probe level, all non-human probes and those not detected by the FES were removed. Only those probes that were detected for at least 50% of the samples in at least one health status group were used for further analysis. Additionally, non-human control features were removed before the analysis. In total, 547 probes, representing 211 miRNAs, fulfilled these criteria. The technical variability of the data was reduced by applying a quantile normalisation [14].

### Genotyping Data Analysis

The single nucleotide polymorphism (SNP) genotype data were generated using Illumina’s HumanOmniExpress array in collaboration with the Institute of Molecular Medicine Finland (FIMM). The chosen array enabled the genotyping of approximately 700k SNPs. To produce the genotype data, the raw data were analysed with Genome Studio according to the manufacturer’s instructions (Illumina, San Diego, USA).

In total, the genotype information for 137 individuals was available, with the miRNA expression levels also measured in 54 of these individuals. Hence, the eQTL analysis was based on these 54 persons. The remaining 83 individuals were used for validation of the results.

### Identification of differentially expressed probes using directional testing

PrCa patients were divided into aggressive (A) and non-aggressive/mild (M) PrCa groups and compared with healthy controls (H). A new generalisation of Mann-Whitney type tests was applied to identify differentially expressed probes in the three-group comparison. The same generalisation was used for the eQTL analysis (for details see [15] and [16]).

For a general definition, let the sample sizes of the three groups be *N*_*H*_, *N*_*M*_ and *N*_*A*_ which results in a total sample size of *N*_*H*_ + *N*_*M*_ + *N*_*A*_ = *N*. The generalised Mann-Whitney test is based on probabilistic indices calculated with triple sums of corresponding indicator functions. Let **x**_*p*;*H*_ = (*x*_1,*p*;*H*_, *x*_2,*p*;*H*_, …, *x*_*N*_*H*_,*p*;*H*_)^*T*^, **x**_*p*;*M*_ = (*x*_1,*p*;*M*_, *x*_2,*p*;*M*_, …, *x*_*N*_*M*_,*p*;*M*_)^*T*^ and **x**_*p*;*A*_ = (*x*_1,*p*;*A*_, *x*_2,*p*;*A*_, …, *x*_*N*_*A*_,*p*;*A*_)^*T*^ be the expression values for a probe *p* in each health group with underlying *cdf*’s *F*_*p*;*H*_, *F*_*p*;*M*_ and *F*_*p*;*A*_. The probabilistic index ${\hat{P}}_{H,M,A;p}$ for probe *p* used in this approach can then be calculated by

$$\begin{array}{c} {\hat{P}}_{H,M,A;p}=\frac{1}{N_{H}N_{M}N_{A}}\sum_{i=1}^{N_{H}}\sum_{j=1}^{N_{M}}\sum_{k=1}^{N_{A}}I(x_{i,p;H}<x_{j,p;M}<x_{k,p;A}), \end{array}$$

and *I*(⋅) is the indicator function that is 1 if condition (⋅) is true and 0 if not. Please notice that the order in the index of ${\hat{P}}_{H,M,A;p}$ refers to the order used in the indicator function.

Furthermore, the probabilistic index ${\hat{P}}_{H,M,A;p}$ can then be used to test the directional hypothesis

$$\begin{array}{c} H_{0}\;:\;F_{p;H}=F_{p;M}=F_{p;A}\;\;\text{vs.}\;\;H_{1}\;:\;F_{p;H}≺F_{p;M}≺F_{p;A}, \end{array}$$

where ≺ refers to the stochastic ordering of *cdf*’s. Naturally, different orders in the condition (⋅) of the indicator function can be used to test for different alternatives. In addition, when expression values are assigned to genotype groups instead of health status, this test procedure is ideal for eQTL testing as it tests for the directional alternatives that are clearly present in the context of an eQTL analysis.

The two probabilistic indices ${\hat{P}}_{H,M,A;p}$ and ${\hat{P}}_{A,M,H;p}$ were used for testing probes *p* = 1, …, 547, and p-values for the permutation test version were calculated based on 5000 permutations. Test results with p-value less than 0.01 were considered to be significant. The test method is implemented in the R-package *gMWT* [16], and the package *GeneticTools* exploits this test method for eQTL testing. Both packages are freely available from the Comprehensive R Archive Network (CRAN).

The Benjamini-Hochberg multiple testing procedure to control the false discovery rate is visualised using rejection plots and lines. The ratio of expected rejections under the null hypothesis is plotted against the observed ratio of rejections. If this curve is above the (0, 1)-line, we have more rejections than expected under the null hypothesis. The rejections for a fixed test size can be visualised with a vertical line, and the rejections for different multiple testing adjustments can be visualised by lines with a certain slope. The number of rejected null hypotheses is then determined by the crossing point of the curve and the line. For details, see [15].

### Classification, Importance Measure and Clustering

The machine learning classifier Random Forest [10], as implemented in the R-package *randomForest* [17], was applied to the expression data, such that the dataset was split into the training (75%) and test (25%) data. The training data were used to create an ensemble of 2500 decision trees, and these trees were then used to classify the test data. The division between the training and validation data was then repeated 2000 times, and afterwards the classification results of all test data runs were evaluated. The Gini importance measure was also extracted for every single Random Forest, and the average importance of each probe was combined with the corresponding p-value from the directional test. Probes that had a p-value less than 0.01 and that belonged to the 10% most important probes over all Random Forest runs were considered to be of high interest (HI probes) and were then used in the clustering step and in the eQTL analysis.

The Random Forests were trained for the three possible outcome classes healthy (H), mild PrCa (M) and aggressive PrCa (A). Let *L*_*i*,*r*;*H*_, *L*_*i*,*r*;*M*_ and *L*_*i*,*r*;*A*_ be the class likelihoods provided by the Random Forest classifier run *r* for individual *i* with *L*_*i*,*r*;*H*_ + *L*_*i*,*r*;*M*_ + *L*_*i*,*r*;*A*_ = 1. These likelihoods were then combined into a single PrCa severeness value $S_{i,r}=\frac{1}{2}L_{i,r;M}+L_{i,r;A}$. The severness value *S*_*i*,*r*_ was chosen in such a way that *S*_*i*,*r*_ = 0 in case that *L*_*i*,*r*;*H*_ = 1, *S*_*i*,*r*_ = 0.5 for *L*_*i*,*r*;*M*_ = 1 and *S*_*i*,*r*_ = 1 if *L*_*i*,*r*;*A*_ = 1.

In a 2-way Random Forest run, the classification was performed only between the healthy and PrCa classes, with same setup as that for the 3-way Random Forest described above.

To calculate the Area Under the Curve (AUC) of the Receiver Operating Characteristic (ROC) curve in the Random Forest case, two different approaches were chosen. First, the two likelihoods *L*_*i*,*r*;*M*_ and *L*_*i*,*r*;*A*_ were added to evaluate the Random Forest’s capability to classify PrCa in general. Then, in the second comparison, the likelihoods *L*_*i*,*r*;*H*_ and *L*_*i*,*r*;*M*_ were added to evaluate its aptitude to identify aggressive PrCa. Eventually, to plot the ROC a continuous cut-off value in [0, 1] was applied onto the likelihood to classify individuals into true/false positives.

For the clustering in the heatmap, the Kendall tau correlation matrix **S** among all samples was calculated based on the expression values of the HI probes. Kendall’ tau between two variables is a measure of positive/negative dependence and is invariant under any strictly increasing transformation to the marginal variables. The corresponding distance between the variables is then defined as **D** = (1 − **S**)/2. Let then **D** be the matrix of distances used for the hierachical clustering.

### eQTL Analysis

**Fig 2:**
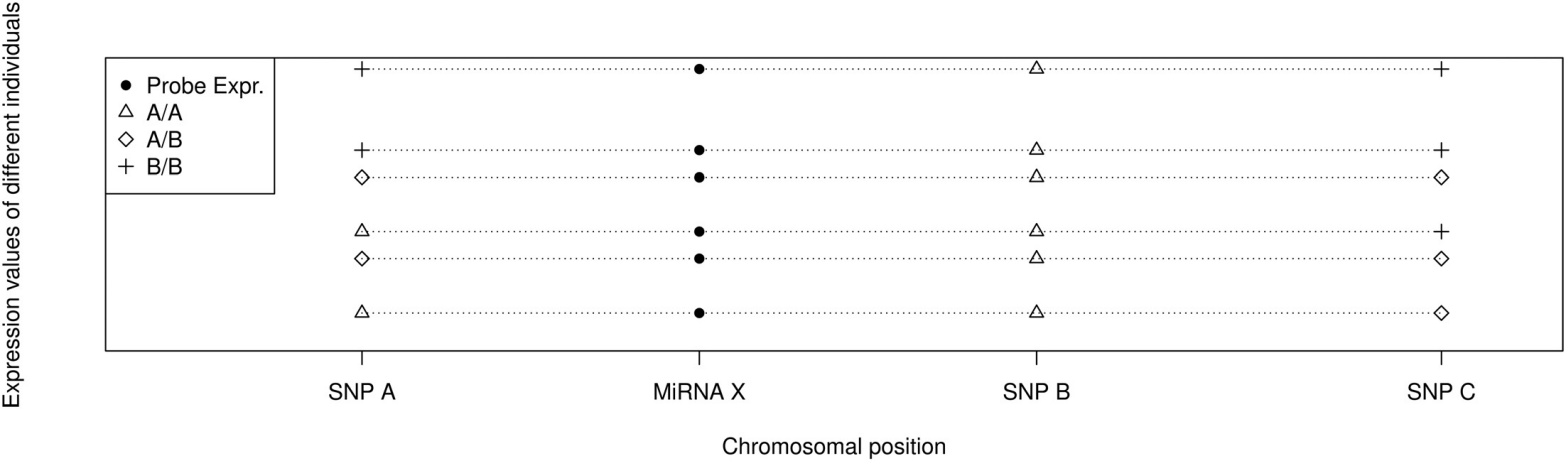
Each line represents an individual, having a certain expression value for miRNA X. Independent of the health status of each individual, the expression values are grouped according to the genotype groups of the surrounding SNPs and then tested for differential expression between those groups. (Figure taken from [16])

The genotype information from the 700k array was combined with the expression values of the HI probes using an eQTL analysis. The chromosomal locations of the miRNA probes were identified and all SNPs within a window of 1Mb around the probe’s central location were linked to this probe. The probe expression values were then assigned to the genotype groups of every linked SNP (Fig 2 shows a systematic sketch of this step).

In an eQTL approach, three cases are possible, depending on whether the expression values have been assigned to one, two or all three possible genotype groups. Monomorphic variants were not further considered in the analysis, and in the two-group case, a two-sided Mann-Whitney test was applied. In the three-group case, the generalised Mann-Whitney test for directional alternatives was used for the two different alternatives whether the higher expression values were linked to the wild-type or the homozygous mutation. This type of directional test was used in the three-group case as an order for the expression values with respect to the genotype groups is clearly expected.

### Comparative Analysis

The here used two-stage approach was compared with two other commonly used methods. The first method was a classical Analysis of Variance (ANOVA), testing the alternative hypothesis that there is a difference between at least two out of the three groups. Let *μ*_*p*,*H*_, *μ*_*p*,*M*_ and *μ*_*p*,*A*_ be the average expression values of probe *p* for the three groups, then is the probe-wise hypothesis for the one-way ANOVA

$$\begin{array}{c} H_{0}\;:\;\mu_{p,H}=\mu_{p,M}=\mu_{p,A}\;\;\text{vs.}\;\;H_{1}\;:\;\text{Not}\;\text{all}\;\mu_{p,.}\;\text{are}\;\text{equal} \end{array}$$

Resulting p-values were then adjusted for multiple testing using a bonferroni correction.

The second method that was used as comparison was a two-staged logistic regression with lasso (LRL). First, LRL was applied onto the full dataset with the two classes healthy/diseased. The tuning parameter *λ* was chosen such that the amount of selected variables were in the same level of magnitude as the here proposed method identifies. The second LRL run was then applied onto the cancer cases only and aimed for the separation of mild and aggressive PrCa. Finally the resulting probes were merged to one result matrix from the LRL analysis.

To compare the results of the ANOVA and the LRL with the here proposed approach, a hierarchical clustering was applied onto the identified probes using also a Kendall’s tau based distance matrix. Then, the adjusted Rand Index was calculated between the classification of the three different clusterings and the true cancer status of the individuals to determine the level of agreement.

## Results

**Fig 3:**
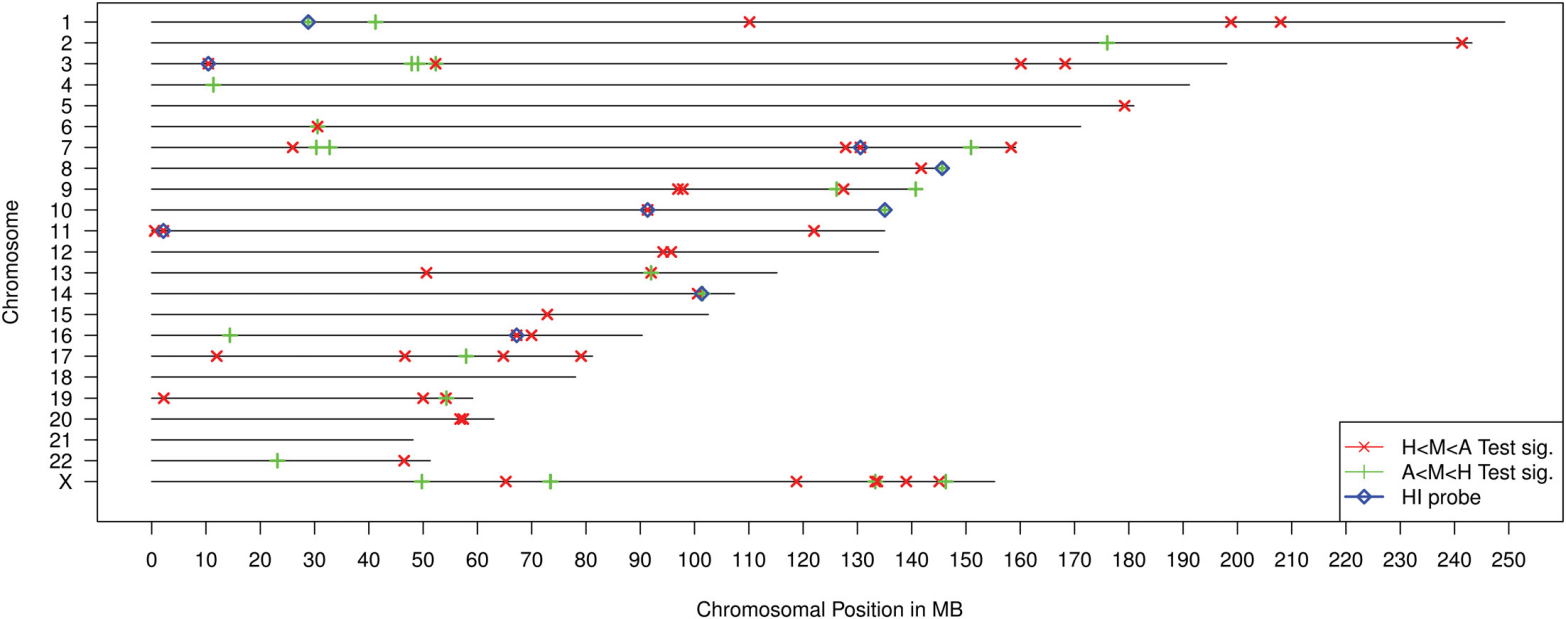
Location of the directional test results for the two probabilistic indicies ${\hat{P}}_{H,M,A}$ and ${\hat{P}}_{A,M,H}$ denoted by *H* < *M* < *A* respective *A* < *M* < *H*. Significant test results that also belong to the 10% most important (Gini Index) miRNAs in the Random Forest run are denoted as HI probes.

Using the directional testing procedure, 146 (87 with higher expression in aggressive PrCa and 59 with higher expression in controls) out of a total of 547 probes were identified having different expression profiles. The chromosomal location of the significant probes and the type of testing alternative are visualised in Fig 3.

To identify HI probes from this unexpectedly large amount of differentially expressed probes, a Random Forest classifier was also applied to the expression data. Significant probes that were within 10% of the most important probes in the Random Forest, measured as Gini Index, were called HI probes and are highlighted in Fig 3. The 13 identified probes represent eight different miRNAs and one spliceosomal RNA. More details about the 13 identified probes are listed in Table 1.

**Table 1:** Overview of the HI Probes, their target miRNAs with corresponding median expression values and chromosomal position.

**ProbeID**	**TargetID**	**ChromosomalLocation**	${\tilde{x}}_{H}$	${\tilde{x}}_{M}$	${\tilde{x}}_{A}$
A_25_P00010263	mir|hsa-miR-328	Chr16:67,236,292—67,236,276	20.81	27.15	25.75
A_25_P00011068	mir|hsa-miR-107	Chr10:91,352,575—91,352,557	405.20	483.35	483.35
A_25_P00011440	mir|hsa-miR-801_v10.1	Chr1:28,847,749—28,847,763	50.77	41.10	34.29
A_25_P00011476	mir|hsa-miR-770-5p	Chr14:101,318,754—101,318,768	28.59	24.52	24.13
A_25_P00011477	mir|hsa-miR-770-5p	Chr14:101,318,755—101,318,768	24.59	20.52	20.53
A_25_P00011979	mir|hsa-miR-770-5p	Chr14:101,318,752—101,318,768	31.16	26.31	26.08
A_25_P00012461	mir|hsa-miR-483-3p	Chr11:2,155,431—2,155,415	24.72	29.56	29.94
A_25_P00012462	mir|hsa-miR-483-3p	Chr11:2,155,431—2,155,414	23.46	29.28	29.35
A_25_P00012991	mir|hsa-miR-885-5p	Chr3:10,436,204—10,436,189	22.43	33.34	32.14
A_25_P00013086	mir|hsa-miR-939	Chr8:145,619,401—145,619,390	152.93	71.98	72.08
A_25_P00013207	mir|hsa-miR-29a*	Chr7:13,0561,530—130,561,511	18.89	21.36	21.52
A_25_P00014864	mir|hsa-miR-202	Chr10:135,061,097—135,061,083	25.92	21.29	20.68
A_25_P00014914	mir|hsa-miR-885-5p	Chr3:10,436,204—10,436,188	21.25	30.92	29.61

**Fig 4:**
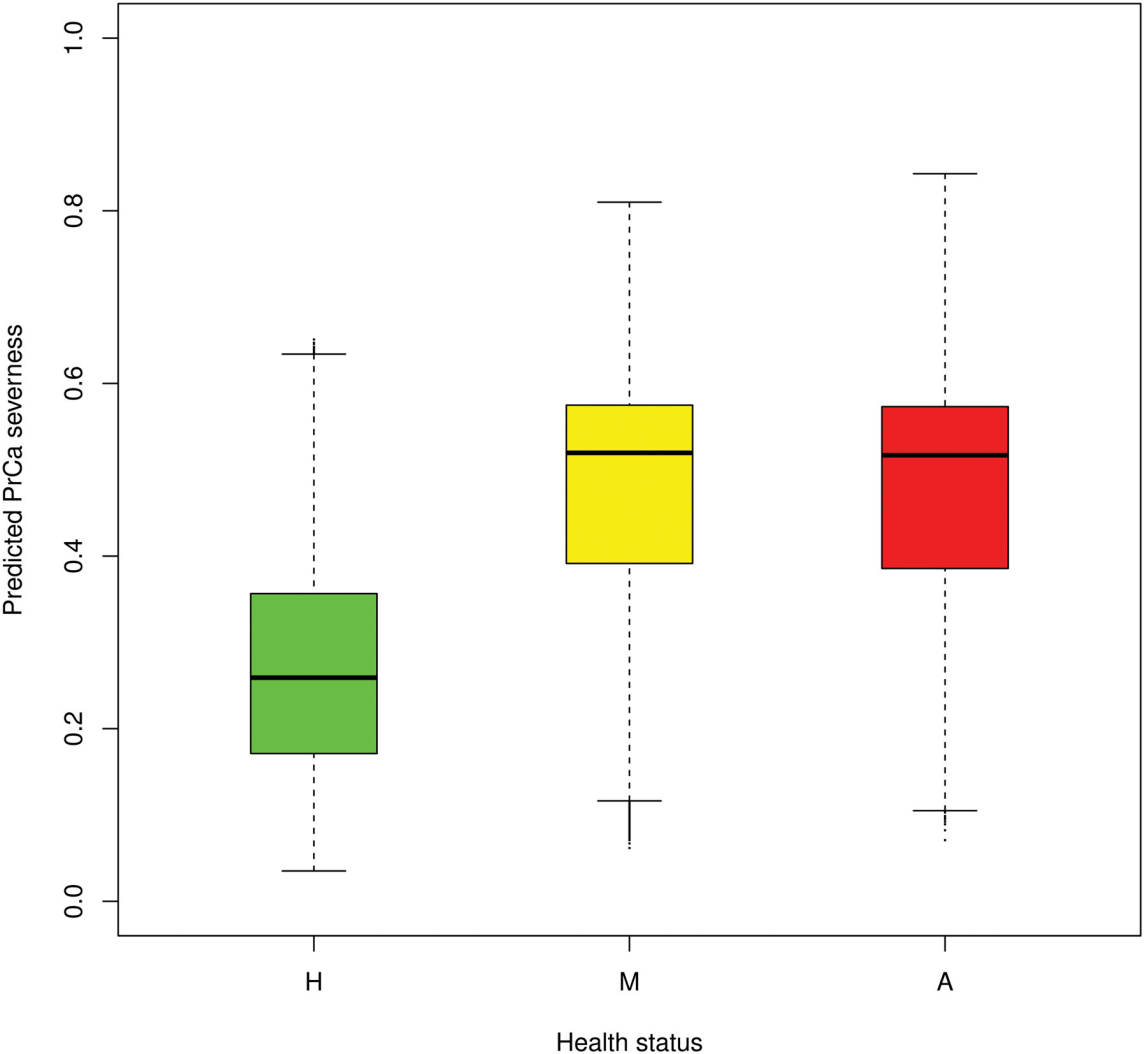
Overall classification results of the Random Forest classifier using the severeness measure *S*_*i*,*r*_.

**Fig 5:**
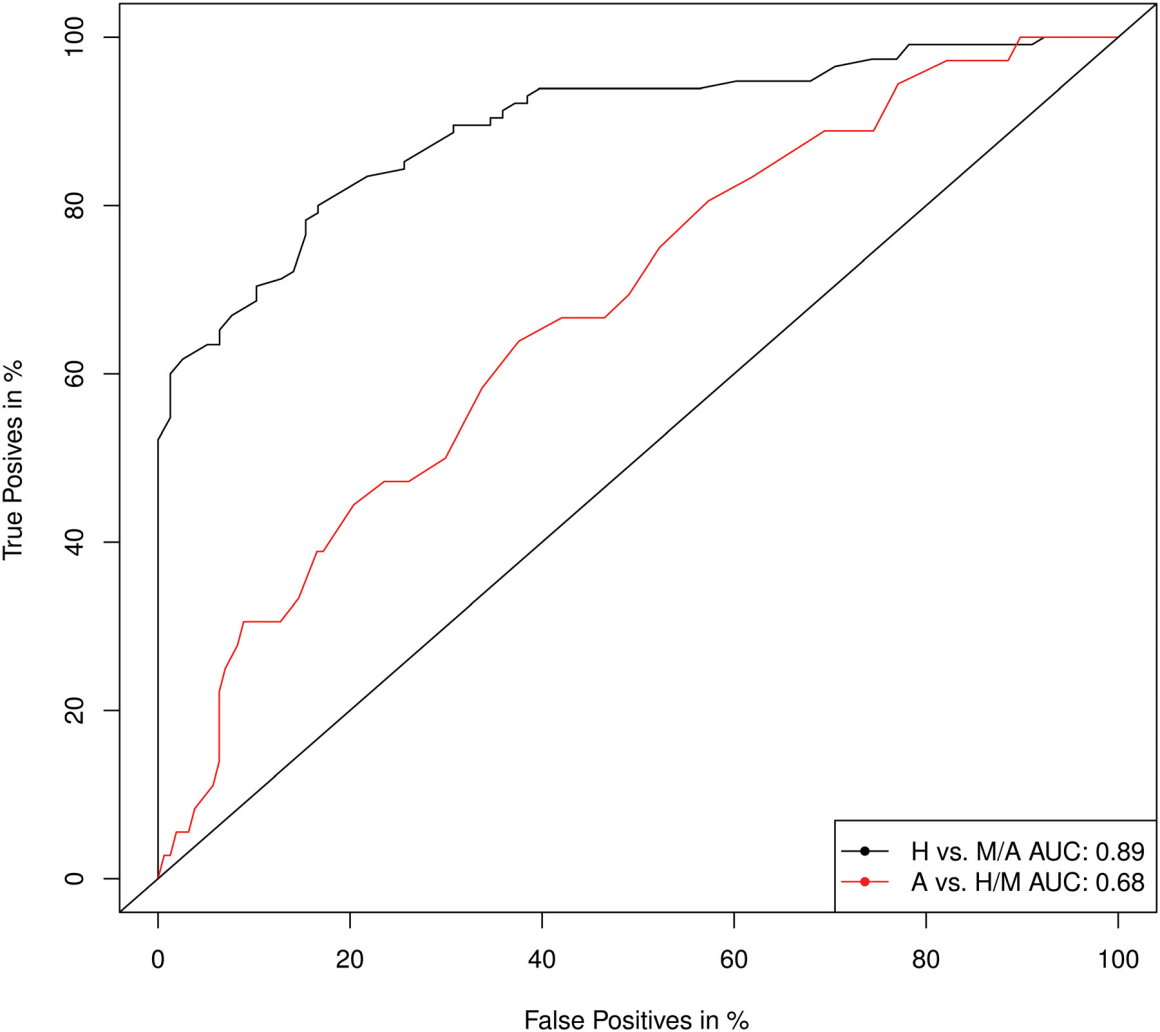
AUCs of different ROCs. Healthy individuals are compared with pooled non-aggressive/aggressive PrCa results (black curve), and aggressive PrCa classifications are compared with the pooled other groups (red).

The overall classification result based on the severeness values *S*_*i*,*r*_ of the Random Forest is visualised in Fig 4. Healthy individuals (green) clearly tended to be in the lower risk area, but aggressive PrCa patients (red) did not tend to have larger values than non-aggressive PrCa patients (yellow). In addition, an average classification rate over all classification runs was determined separately for the comparisons between healthy and PrCa and between aggressive PrCa and combined healthy and non-aggressive PrCa. The Random Forest was able to classify PrCa with an average AUC of the ROC of approximately 0.89 and aggressive PrCa versus the combined samples of non-aggressive PrCa and controls of 0.68 (Fig 5). The classification results at the individual level are visualised in the supporting information (S1 and S2 Figs).

**Fig 6:**
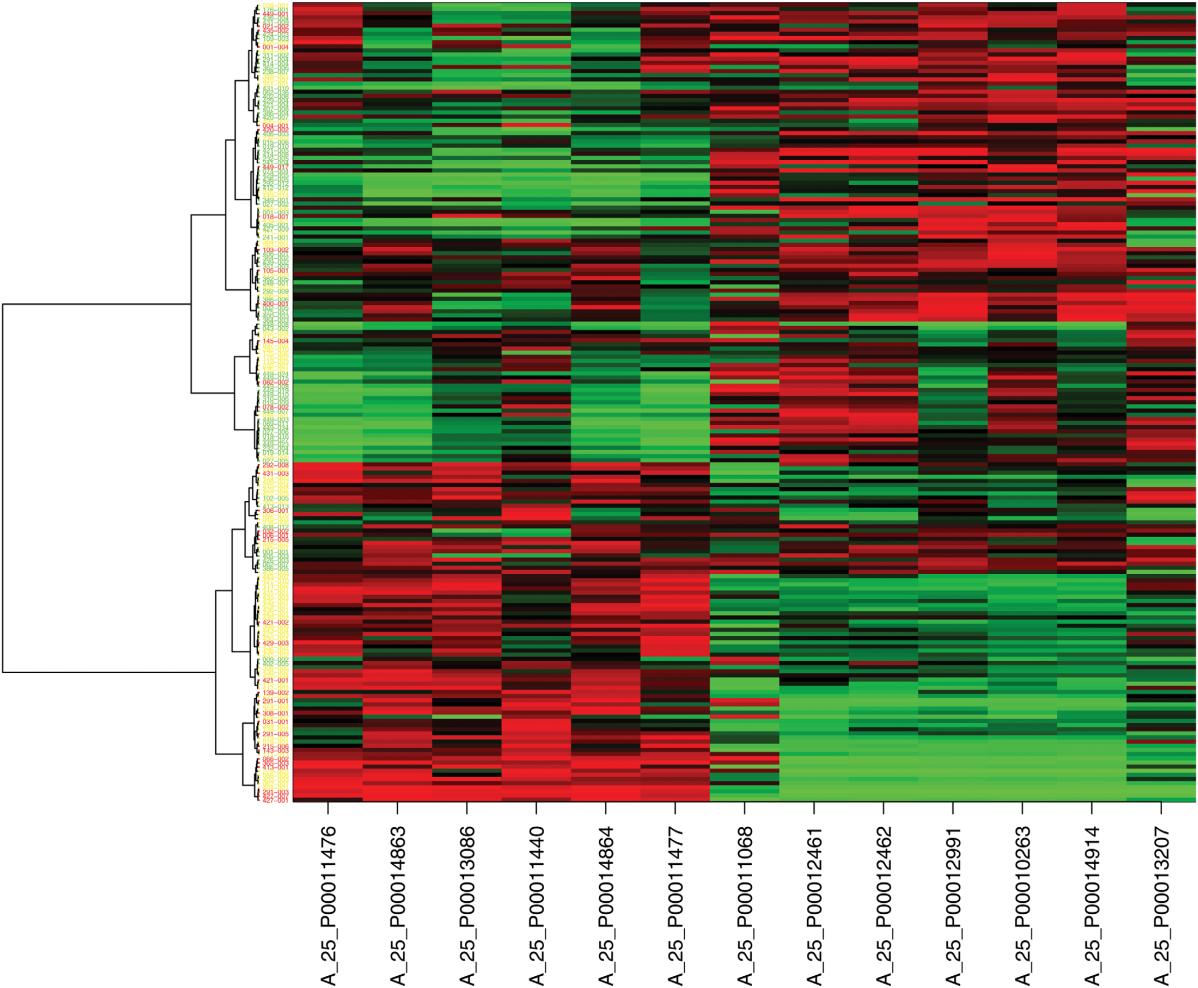
Heatplot of the HI probes. Red colours refer to low expression values, whereas green colours represent large expression values for particular probe. The miRNA targeted IDs corresponding to the given probe IDs are listed in Table 1. Colors in the dendrogram represent the observed health status (green: healthy, yellow: non-aggr. PrCa, red: aggr.PrCa)

A hierarchical clustering shows the importance of the HI probes. Clustering the dataset based on all probes resulted in only a slightly better classification than the clustering based on the 13 HI probes. The dendrogram for clustering individuals based on the 13 HI probes together with the corresponding heatmap is shown in Fig 6. Here, the ability to separate clearly between aggressive and non-aggressive PrCa was limited, but interestingly only five of the 78 healthy individuals were clustered closely together with PrCa individuals. In contrast, 46 of 115 PrCa cases were inside the cluster that contained most of the healthy individuals.

In addition, a cis-eQTL (0.5Mb up/downstream window) for the HI probes was performed. In total, 3863 SNP-miRNA associations were tested, and 79 had a p-value of ≤ 0.01, (S3 Fig in the supporting information). All SNPs that were found to have a possible regulatory effect on an HI probe were then tested for a direct PrCa association by applying a Fisher-test on the 2 × 3 table between genotype and health status groups. For four SNPs, a significant association was found for the 53 genotypes of the eQTL samples (test size 0.05).

In the samples for which only genotype data was available, six associated SNPs were found, but significant SNPs from the first, initial test could not be validated with the additional genotype data. For both data sets however, there were one, respective four (out of 15) significantly associated SNPs in cis-location of miRNA hsa-miR-483-3p (see Table 2 for detailed information).

**Table 2:** SNPs associated with miRNA expression levels that were also shown to be directly associated with PrCa using a Fisher-test with a significance level of 0.05. The upper part is from the eQTL dataset, and the lower part is the results for the validation data.

			**Allele**		**Freq**		
**SNP**	**Mirna in *cis***	**Location**	**1**	**2**	**PrCa**	**H**	**P-Value**
RS547311	mir|hsa-miR-29a*	Chr7:130,598,454	G	A	32;4;3	8;6;1	0.0410
RS17173843	mir|hsa-miR-483-3p	Chr11:1,679,711	G	A	30;9;0	8;5;2	0.0433
RS11849923	mir|hsa-miR-770-5p	Chr14:101,138,605	A	G	18;16;5	2;12;1	0.0309
RS11629195	mir|hsa-miR-770-5p	Chr14:101,328,739	A	G	3;24;12	5;3;7	0.0084
RS7079873	mir|hsa-miR-107	Chr10:91346003	A	C	62;1;0	16;4;0	0.0110
RS7913785	mir|hsa-miR-107	Chr10:91348352	A	C	62;1;0	17;3;0	0.0417
RS4752755	mir|hsa-miR-483-3p	Chr11:1756848	A	G	18;30;15	9;11;0	0.0270
RS1317356	mir|hsa-miR-483-3p	Chr11:1779138	A	G	17;30;16	9;11;0	0.0149
RS756919	mir|hsa-miR-483-3p	Chr11:2310605	A	C	52;11;0	11;7;2	0.0115
RS1501466	mir|hsa-miR-483-3p	Chr11:2320884	A	G	41;21;1	14;3;3	0.0226

Finally the here proposed method was also compared to an ANOVA approach and a LRL. Using a multiple testing adjusted significance level *α* = 0.001 resulted in 14 significant probes, whereas the LRL tuning parameter was set such, that LRL identified 15 probes to be of high interest. The amount of intersecting probes between these two approaches was seven, whereas the intersection of the HIprobes with the ANOVA probes was just five and with LRL even only three. Comparing the quality of the clustering based on those probes using the Adjusted Rand Index, resulted in a Rand Index of 0.168 for the probes identified by the here proposed approach, 0.130 for the ANOVA and 0.131 for the LRL approach.

## Discussion

The aims of the study were to apply novel statistical methods that better differentiate aggressive from indolent prostate cancer and, are robust against outliers and to survey the prognostic and diagnostic values of blood-derived miRNAs.

In this study, we used a generalised Mann-Whitney approach [15] combined with the Random Forest algorithm to identify differentially expressed miRNAs. By combining the two methods, we were able to significantly reduce the panel of interesting miRNAs. The advantage of this approach is that it effectively combines the two different methods to detect meaningful variables. Each approach by itself identified a large number of significant miRNAs, even after controlling the false discovery rate. However, combining these two methods provided a shorter list of miRNAs of potential interest, reducing effectively the amount of false positive test results. S4 Fig in the supporting information shows details about the test rejections and the consequences of a Benjamini-Hochberg correction.

Without any multiple testing correction, the both tests showed rejection rates of approximately 16% and 10% for a test size of 0.01. Accepting a false discovery rate of 0.05% the rejection rates were still in the order of 5–10%. Instead of controlling just the false discovery rate, a multiple testing method was omitted, and an ensemble method that combines the results of the two different approaches was used instead. Although this was done on a possible expense of many false negative test results, the here identified set gained further confidence by combining the test results.

In addition to the development of analytical tools, obtaining good matches between cases and controls is important, especially in miRNA studies for which the findings among studies are often conflicting. The use of Finnish familial PrCa cases and their healthy relatives enabled to reduce the background heterogeneity of the miRNA expression profiles to be reduced. In fact, individuals within families were observed to share a miRNA signature specific for the family, and family members were more often clustered next to each other. Consequently, informative miRNA biomarkers that can distinguish patients from their healthy counterparts within one family are extremely interesting.

Altered miRNA expression has been identified in different malignancies. Depending on the expression profile in the tumour, they can act as either oncogenes or tumour suppressors. Our protocol identified eight miRNAs and one splicosomal RNA with potential importance in determining the PrCa risk. Five of the miRNAs (miR-483-3p, miR-29a, miR-107, miR-885-5p and miRNA-328) were upregulated, which most often has been suggested to be associated with an oncogenic role. Two of these miRNAs have been previously associated with PrCa.

For miR-483-3p an upregulated and oncogenic role has been previously reported in Wilm’s tumours and colon, breast and liver cancers [18] due to its antiapoptotic role. This antiapoptotic function was shown to be mediated through interactions with the 3’UTR of PUMA mRNA—(p53 upregulated modulator of apoptosis/BCL2 binding component 3), a proapoptotic protein inhibiting the antiapoptotic factors BCL3 and BCLXL [19]. Moreover, the location of miR-483-3p in chromosomal region 11p15.5 is interesting as the region is known for genetic aberrations and epigenetic abnormalities, including imprinting [20] [21]. Here, additional possible regulatory SNPs in cis of mir-483-3p were found through the eQTL analysis, with five of those SNPs (rs17173843, rs4752755, rs1317356, rs756919 and rs1501466) also identified as being directly associated with PrCa risk in the validation tests.

Another interesting microRNA upregulated in the familial PrCa cases was miR-29a, which was recently reported to be upregulated in different cancers [22]. MiR-29a is a member of the miR-29 family, which mainly regulate the same target genes. The biological functions of miR-29s mediate several tumour-related pathways, including cell proliferation, apoptosis and metastasis. MiR-29a has recently been reported to be regulated by c-Myc and CCAATT/enhancer-binding-protein-(CEBPA), two genes that have been recognised as important modulators in multiple cancers, also in PrCa.

The third significantly up regulated microRNA in cancer patients in our study was miR-107. A recent report identified an oncogenic role for miR-107 that is mediated by the negative regulation of tumour suppressor miR let-7 [23]—which decreased expression of miR-let-7 has been reported in PrCa [24]. In addition, its role in the aggressiveness of PrCa has been proposed to be mediated through its interaction with the Enhancer of Zeste homolog 2 (EZH2), the over-expression of which is strongly linked to cancer progression [25].

The fourth upregulated miRNA in our analysis was miR-885-5p, which potential as a marker has been previously proposed in liver pathologies and gastric cancer. MiR-885-5p was upregulated in cases and had an AUC of 0.904 with 90.53% sensitivity and 79.17% specificity to discriminate liver pathologies from healthy controls [26]. Controversially, tumour suppressive role has been suggested for miR-885-5p in neuroblastoma, where it interferes with cell proliferation and survival through cyclin-dependent kinase (CDK2), minichromosome maintenance protein (MCM5) and p53 [27].

In the present study, miRNA-328 was also upregulated in PrCa and was one of the most important contributors in our clustering procedure. In addition to complementary mRNA interference, this miRNA has been proposed to have a role in decoy activity [28].

Together with the five upregulated miRNAs, three down regulated miRNAs (miR-939, miR-202 and miR-770) and the U11 spliceosomal RNA (RNU11, U11 snRNP or mir-801) were observed. For all but miR-770, cancer related pathways have been suggested. The loss of endogenous miR-939 function has been reported to increase iNOS (inducible nitric oxide synthase) protein in hepatocytes [29]. Increased iNOS expression, in contrast, has been associated with poor outcome/survival in breast cancer and melanoma [30] [31]. In PrCa, high iNOS expression has been associated with high pT classification and high preoperative PSA [32].

The target analysis of miR-202 in HeLa cells revealed two interesting targets, -DICER1 and SKP2, with potential importance in cancer [33]. The down regulation of miR-202 would potentially lead to the upregulation of the DICER1 protein. DICER1 is a key mediator of miRNA processing that, when impaired, accelerates cell transformation and tumorigenesis [34]. The down regulated U11 snRNP has been reported to interact with components of the U2-dependent spliceosome and function as an activator of alternative splicing [35].

This mechanism has an important role in regulation of protein diversity and many reports indicate cancer-associated changes in alternative-splicing [36] [37] [38].

The used study protocol has possible shortcomings. As the expression of many miRNAs is tissue-specific, a study protocol with matched peripheral blood and tumour tissue samples would be ideal for all analyses. However, from the perspective of a diagnostic method, blood samples are preferred tissues for analytical purposes. Herein, the use of EBV (Epstein-Barr virus)-transformed LCLs has to be taken into account. The use of LCLs in genetic studies is common, and the concordance of mRNA expression between EBV-transformed LCLs and other cell types has been reported [39] [40]. Serum or plasma miRNAs may indicate the presence of a tumour, but the question remains whether the ncRNAs that are differentially expressed in the LCLs of matched cases and controls truly represent the mechanism of increased cancer predisposition.

For the analysed samples, the cancer status is updated from the Finnish Cancer Registry on a regular basis. Consequently, some of the sampled individuals who were healthy at the time of the blood-draw later developed PrCa. Therefore, in our analysis, with knowledge of this change, these individuals were now labelled as PrCa cases. The PrCa classification status was adjusted in this manner for a total of 19 individuals. In the testing sets of the 2-way Random Forest classification, these 19 individuals had an average severeness value of 0.76, with only one of them having an average value less than 0.5. This finding indicates that the miRNA profiles of the 19 individuals were already different from those of healthy individuals, although PrCa had not yet been diagnosed. In the heatmap clustering, five of these individuals were in the so-called healthy cluster, indicating the advantage of the Random Forest over the classic clustering based on a correlation measure.

For these 19 individuals, the difference between the blood-draw and the diagnosis dates was an average 3.2 (ranging from 0.1 to 9.6) years. Their average PSA value at diagnosis was 8 (ranging from 0.8 to 19.7). Three individuals have died already (4.7, 8.8 and 16.1 years after diagnosis), although PrCa was not confirmed as the reason for death.

Likewise, there are 11 healthy controls that had a severeness value greater than 0.6 in the Random Forest analysis. One of these individuals was labelled as healthy although the Cancer Registry data later revealed that, he suffered from bladder cancer and eventually died of this disease. For the remaining 10 healthy individuals, no known, cancer-related explanation for the misclassification could be found. For this group of individuals, the current median age is now 69.3 (ranging from 56.4 to 94.2) years. In addition there were nine false negative classifications in the Random Forest test data (with severeness values less than 0.4). Four of these individuals are deceased, but only one was confirmed to have died from PrCa (age at diagnosis: 73.5 years, age at death: 87.9 years). The remaining three patients died on average approximately 10 years after diagnosis due to other reasons (one patient) or unknown reasons (two patients). The remaining five false negative cases have had PrCa for 7–22.4 years, possibly indicating that they do not have an extremely aggressive clinical outcome. Assuming a threshold of 0.5, there would have been 21 false positives, and the same nine false negative individuals would have still been detected. However, due to the variance in the classification introduced by the setup of the Random Forest, selecting 0.4 and 0.6 as the thresholds for false positives and negatives, respectively, and maintaining the region [0.4, 0.6] as undecided, appeared to be reasonable.

In this kind of genetic studies, many false positive test results can be expected to occur. Given this knowledge, we aimed for a smaller number of significant results to potentially reduce the risk. It was surprising that even after a multiple testing correction, many significant results were detected by our directional testing method. A comparison with traditional methods such as ANOVA or Logistic Regression with Lasso showed, first, that the here proposed method identified a different set of probes to be of high interest and, second, that the identified probes have even a better capability of classification during the clustering. However, when LRL was applied using a tuning parameter based on cross-validation its classification capabilities during clustering were slightly better than the here proposed method (Rand Index 0.19), but for the price of identifying twice as many probes.

Our approach of combining the test results from a non-parametrical, directional test with the importance measure of the Random Forest method, however, was able to further reduce the number of interesting probes. The respective miRNAs of detected probes have all been previously reported to be interesting in a tumour context. These findings strongly indicate that the combination of the results is meaningful.

In conclusion, in our study we were able to identify a reasonable panel of differentially expressed miRNAs from the LCLs of hereditary PrCa patients and their healthy relatives. The relevancy of the detected miRNAs encourage the further use of LCLs to detect altered miRNA expression as a risk factor for cancer. However, further studies are needed to identify the pathways in which these miRNAs are involved. Moreover, learning the processes and underlying biology that determine the effect LCL-based miRNAs on PrCa predisposition and development is particularly important.

We do not propose that the here presented two-step approach as a superior tool over traditional methods, but rather suggest it as a new tool in the analysis toolbox. However, when applying it onto the dataset at hand, it showed promising results and hence its trial on other datasets is warranted. The concept is similar to diagnostic testing, where several complementary tests are used instead of only one.

## Supporting Information

S1 Fig Random Forest classification results for the classification of healthy (green), non-aggressive PrCa (yellow) and aggressive PrCa (red) using a Random Forest for three groups. (EPS)

S2 Fig Random Forest classification results for the classification of healthy (green), non-aggressive PrCa (yellow) and aggressive PrCa (red) based on a binary Random Forest that was trained for the Healthy/PrCa outcome only. (EPS)

S3 Fig Location of the significant SNPs for the HI probes. (EPS)

S4 Fig Rejection plot of the direction test. The green dotted line indicates the chosen test size of 0.01 and there approximately 16% for the test H < M < A, respective 10% for the A < M < H being rejected. The blue dotted line indicates the rejection if we had performed a Benjamini-Hochberg multiple-testing adjustment. There would be still a rejection rate of 10% respective 5% of all miRNAs.
(EPS)
